# Transgenic overexpression of CTRP3 does not prevent alcohol induced hepatic steatosis in female mice

**DOI:** 10.1371/journal.pone.0258557

**Published:** 2022-01-07

**Authors:** Kristy L. Thomas, Callie L. Root, Jonathan M. Peterson

**Affiliations:** 1 Department of Health Sciences, East Tennessee State University, Johnson City, TN, United States of America; 2 Department of Biomedical Sciences, Quillen College of Medicine, East Tennessee State University, Johnson City, TN, United States of America; Johns Hopkins University School of Medicine, UNITED STATES

## Abstract

Alcoholic liver disease (ALD) is one of the leading causes of morbidity and mortality from hepatic complications. C1q/TNF-related protein 3 (CTRP3) is an adiponectin paralog and, in male mice, increased levels of circulating CTRP3 prevents ALD. Therefore, the purpose of this study was to replicate the observed hepatoprotective effect of elevated circulating CTRP3 levels in female mice. Twelve-week-old female wildtype and CTRP3 overexpressing transgenic mice were fed the Lieber-DeCarli alcohol-containing liquid diet (5% vol/vol) for 6 weeks. Unlike the previous study with male mice, CTRP3 overexpression provided no attenuation to alcohol-induced hepatic lipid accumulation, cytokine production, or overall mortality. In conclusion, there appears to be a clear sex-specific effect of CTRP3 in response to alcohol consumption that needs to be explored further.

## Introduction

Chronic liver disease is the 12^th^ leading cause of morbidity and mortality worldwide [1–8]. Almost half of liver-related deaths are due to alcohol over-consumption [6], referred to as alcoholic liver disease (ALD). The two major causes of excess lipid buildup in the liver result from a high-fat diet (referred to as nonalcoholic fatty liver disease (NAFLD) and/or overconsumption of alcohol, ALD. Morphologically, both NAFLD and ALD have similar histopathological structures [8–10]. Hepatic steatosis or fatty liver results from excess lipid storage in the liver (lipids > 5% liver weight) [10]. Overconsumption of alcohol causes changes in the liver’s metabolism; lipid catabolism decreases while lipogenesis and lipid mobilization increase, leading to the accumulation of lipids in the liver [10]. Alcoholic hepatic steatosis is mild and reversible; however, an individual may not be aware they have it, and without interventions can progress into alcoholic hepatitis, liver fibrosis, and cirrhosis, and ultimately into alcoholic hepatic failure [4, 11]. Current interventions to treat hepatitis from ALD include abstinence from alcohol, corticosteroids, and nutritional support [4, 7]. Further, although the amount of alcohol consumption is generally related to the severity of the disease, the reasons for the progression of ALD to chronic hepatitis and alcoholic cirrhosis in subset of patients are unknown.

Adipose tissue is not only a primary tissue designed for the long-term storage of excessive lipids, adipose tissue also secretes a number of biologically active molecules, which circulate through the blood, named adipokines [4, 11–16]. Adipokines play a role in energy metabolism, inflammation, and survival signaling and are dysregulated in metabolic disease or with excessive alcohol consumption [4, 17]. A relatively new group of adipokines called C1q/tumor necrosis factor-related proteins (CTRPs) have been identified [16, 18]. C1q TNF-related protein-3 (CTRP3, also known as Collagenous repeat-containing sequence 26 kDa protein, cartducin, or cartonectin) is of particular interest as elevated CTRP3 has been demonstrated to prevent diet-induced fatty liver (NAFLD) and individuals with NAFLD have reduced circulating level of CTRP3 [19]. Even though the mechanism has yet to be determined, these data show that CTRP3 could be a potential therapeutic to treat ALD in the future.

Trogen et al. [20] determined transgenic overexpression of CTRP3 in male mice is protective against long-term lipid accumulation from alcohol consumption; females were not tested. Since the 1970’s it has been observed that alcoholic cirrhosis occurs at a higher rate in female alcoholic patients compared with males, at an earlier age, with a lower proportional amount of alcohol consumption [8, 11, 17, 21–29]. Although there are 2–3 times more total deaths resulting from liver failure due to alcohol consumption in men than in women, there are 4–5 times more men considered heavy drinkers compared with women [2, 4–6, 9]. Further, Degroat et al. [17] observed that circulating CTRP3 levels were specifically reduced in alcohol-consuming female mice. Therefore, the purpose of this project was to build on the previous study and test the hypothesis that female mice would also be protected by overexpression of CTRP3 in alcohol-induced ALD.

## Methods

### Animals

The CTRP3 transgenic overexpression (Tg) mouse strain was developed on the C57Bl/6J background with the carboxy-terminal FLAG epitope (DYKDDDDK)-tagged CTRP3 expression driven by the CMV early enhancer/chicken-actin (CAG) promoter as described previously [19]. Female mice were pair housed in polycarbonate cages on a 12:12-h light-dark photocycle with ad libitum access to water and food, except as specified. Littermates without the CTRP3 transgene were used as wild-type (WT) controls. Throughout the experimental protocols, mice were inspected and weighed daily. At the time points indicated, animals were anesthetized with isoflurane and euthanized via exsanguination. Serum samples were collected and prepared according to the manufacturer’s instructions (Sarstedt, cat. no. 41.1500.005). Tissue samples were excised and the left lobe of the liver was fixed in 10% formalin for histology with the remaining tissue snap-frozen in liquid nitrogen, and stored at -80°C until further analysis. All animal procedures were conducted in accordance with institutional guidelines, and ethical approval was obtained from the East Tennessee University Committee on Animal Care (Animal Welfare Assurance no. A3203-01).

### Chronic ethanol feeding

12-wk-old female mice were acclimatized to a liquid diet *ad libitum* without the addition of alcohol for 1-wk and then gradually transitioned from 1 to 5% Lieber-DeCarli ethanol diet (*vol/vol* ethanol) over the course of the next 2 weeks and then maintained with 5% ethanol (vol/vol ethanol) for the remaining 4 weeks. Food intake was measured daily for each cage (2 mice per cage), and body weight was recorded for each individual mouse daily. Animals were euthanized and counted as dead based on the presence of any of the following criteria: unconsciousness with no response to external stimuli, intractable seizures, labored breathing or respiratory distress, inability to ambulate or maintain upright position, chronic or debilitating vomiting, diarrhea or constipation, or decrease in body weight >10% week-to-week, beginning after the week 2 of the feeding protocol. On the morning of the final day, food was removed, and mice were fasted for 9 h before euthanasia.

### Liver histology

A section of the left lobe of the liver was fixed in 10% formalin for 2 days. Following fixation, the tissue was dehydrated and embedded in paraffin using standard embedding techniques. Each sample was sectioned at 5 μm, mounted, and stained using standard hemotoxylin and eosin (H&E) staining procedures. The tissue was examined and 20x images were photographed using a Zeiss Axioskop 40 microscope. Stereological analysis was performed manually on three non-overlapping images (20x) from each tissue section via a 100-point grid overlay. Each point was then identified as hepatic, extra-hepatic, or lipid droplet.

### Hepatic lipid analysis

Lipids were extracted as described by Bligh and Dyer and as previously performed [19, 20, 30]. Briefly, liver samples were weighed and homogenized in phosphate-buffered saline (10 ml/g tissue), followed by the addition of 1:2 (vol/vol) chloroform-methanol (3.75 ml/ml sample homogenate). Next, chloroform was added (1.25 ml/ml sample homogenate), followed by a final addition of distilled water (1.25 ml/ml sample homogenate). Samples were vortexed for 30 s between each step. Samples were then centrifuged (1,100 *g* for 10 min at room temperature) for phase separation (aqueous phase on top and organic phase below). The lower phase was collected with a glass pipette with gentle positive pressure (so as not to disturb the upper phase). Samples were then divided into two aliquots and dried under nitrogen gas at 60°C. Each sample was dissolved in *tert*-butyl alcohol:Triton X-100 (3:2 vol/vol) solution. Triglycerides were quantified via colorimetric assay according to the manufacturer’s directions (Fisher Diagnostics, cat. no. TR22421).

### Biochemical and hormonal variables

Serum triglyceride concentrations were determined using commercially available assays according to the manufacturers’ directions (Fisher Diagnostics, cat. no. TR22421). Interleukin-6 (IL-6), interferon gamma (IFN-γ), interleukin-1 beta (IL-1β), interleukin-10 (IL-10), interleukin-17 alpha (1L-17α) and tumor necrosis factor-α (TNFα) were determined with commercially available kits using the Bio-Plex Multiplex Immunoassay System (Bio-Rad cat. nos.171G5007M, 171G5017M, 171G5002M, 171G5009M, 171G5013M, 171G5023M). The intra- and inter-assay coefficients of variation for all assays were 8% and 11%, respectively. Any value below the detectable assay working range was assigned the value equivalent of the lower limit of quantification.

### Immunoblot analysis

Liver samples were powdered in liquid nitrogen, and proteins were extracted from ~100 mg suspended in radioimmunoprecipitation assay (RIPA) buffer (50 mM Tris·HCl, pH 8.0, 150 mM NaCl, 0.1% Triton X-100, 0.5% sodium deoxycholate, 0.1% SDS) with protease and phosphatase inhibitors (Bimake, cat. nos. B14001 and B15001). The samples were incubated for 30 min at 4°C with gentle rotation and then centrifuged at 16,000 *g* for 10 min at 4°C. The supernatant containing the soluble fraction was collected and prepared for protein quantification. Protein concentrations were determined by commercial assay according to the manufacturer’s directions (Thermo, cat. no. 23238). Equal concentrations of proteins were separated by SDS-polyacrylamide gel electrophoresis in SDS loading buffer (4% SDS, 10% 2-mercaptoethanol, 20% glycerol, 0.004% bromophenal blue, 0.125 M Tris·HCl, pH 6.8), according to the manufacturer’s directions (Bio-Rad, cat. no. 456–1046). Equal loading was confirmed by Ponceau S stain (0.1% Ponceau S, 5% acetic acid). Membranes were blocked with 2% nonfat milk solution for 1 h at room temperature and then probed with primary antibodies (Table 1) overnight at 4°C, followed by appropriate horseradish peroxidase (HRP)-conjugated secondary antibody (Table 1). Chemiluminescent signals were detected (chemiluminescent HRP substrate, Millipore, cat. no. WBKLS0100) and quantified using the Bio-Rad ChemiDoc Imaging System and Bio-Rad ChemiDoc Software. Band intensities were measured densitometrically and expressed as fold changes relative to those of normal control livers. Full ponceau red staining and immunoblots images are attached as supplemental data (S1 Fig).

**Table 1:** Antibodies.

Protein	Source	RRID
pPKB (Ser^437^)	Cell Signaling Technology cat. no. 4051	AB_331158
PKB	Cell Signaling Technology cat. no. 4685	AB_2225340
p-MAPK 1/3 (Thr^202^/Tyr^204^)	Cell Signaling Technology cat. no. 4370	AB_2315112
MAPK 1/3	Cell Signaling Technology cat. no. 9102	AB_330744
Rabbit IgG, HRP	Thermo Fisher Scientific cat. no. 31460	AB_228341
Mouse IgG, HRP	Thermo Fisher Scientific cat. no. G-21040	AB_330924

PKB, protein kinase B; p-, phosphorylated; MAPK 1/3, mitogen-activated protein kinase; GAPDH, glyceraldehyde 3-phosphate dehydrogenase; HRP, horseradish peroxidase-conjugated secondary antibodies; RRID, Research Resource Identifier (http://scicrunch.org/resources).

### Statistical analysis

Descriptive statistics (means and SDs) were calculated for all measured variables. Body weight and food intake were analyzed by two-way repeated-measures ANOVA followed by Sidak’s multiple comparisons test. Survival curve was analyzed by the Log-rank (Mantel-Cox) test. An unpaired *t*-test was used to compare all remaining data between CTRP3 Tg and WT. All statistical analyses were performed with GraphPad Prism 9.

## Results

### CTRP3 overexpression impacts on food intake and body weight

**Fig 1:**
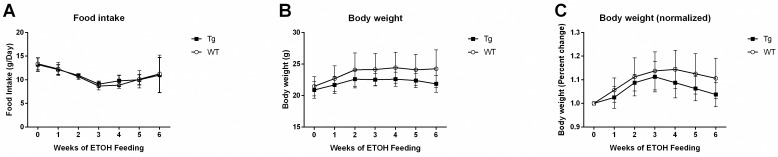
Animal characteristics. A) Daily measurements of food intake were reported as diet consumed in grams per mouse each day. Daily body weights were also reported as both raw values (B) as well as normalized to starting weight (C). Data reported as means ± SD; n = 8 and 16, Tg and WT respectively. Data were compared with a repeated measures ANOVA followed by Sidak’s multiple comparisons test. Abbreviations: CTRP3, C1q tumor necrosis factor-related protein-3; Tg transgenic overexpression of CTRP3; WT, Wildtype.

There was no difference in food intake between the Tg and WT mice (Fig 1A). While it was expected for the mice to lose up to 10% of their body mass during the first week of transition to ethanol containing diet, no loss of body mass was observed (Fig 1B, 1C). Further, there was no significant difference in body weight throughout the duration of the ethanol feeding between the WT and Tg mice. Body weight and food intake data from mice that were found dead or culled prior to week 6 were not included (1 Tg mouse and 2 WT mice). Circulating CTRP3 levels in wildtype compared with transgenic male and female mice are attached as supplemental data (S2 Fig).

### CTRP3 overexpression did not improve survival in response to ethanol feeding in female mice

**Fig 2:**
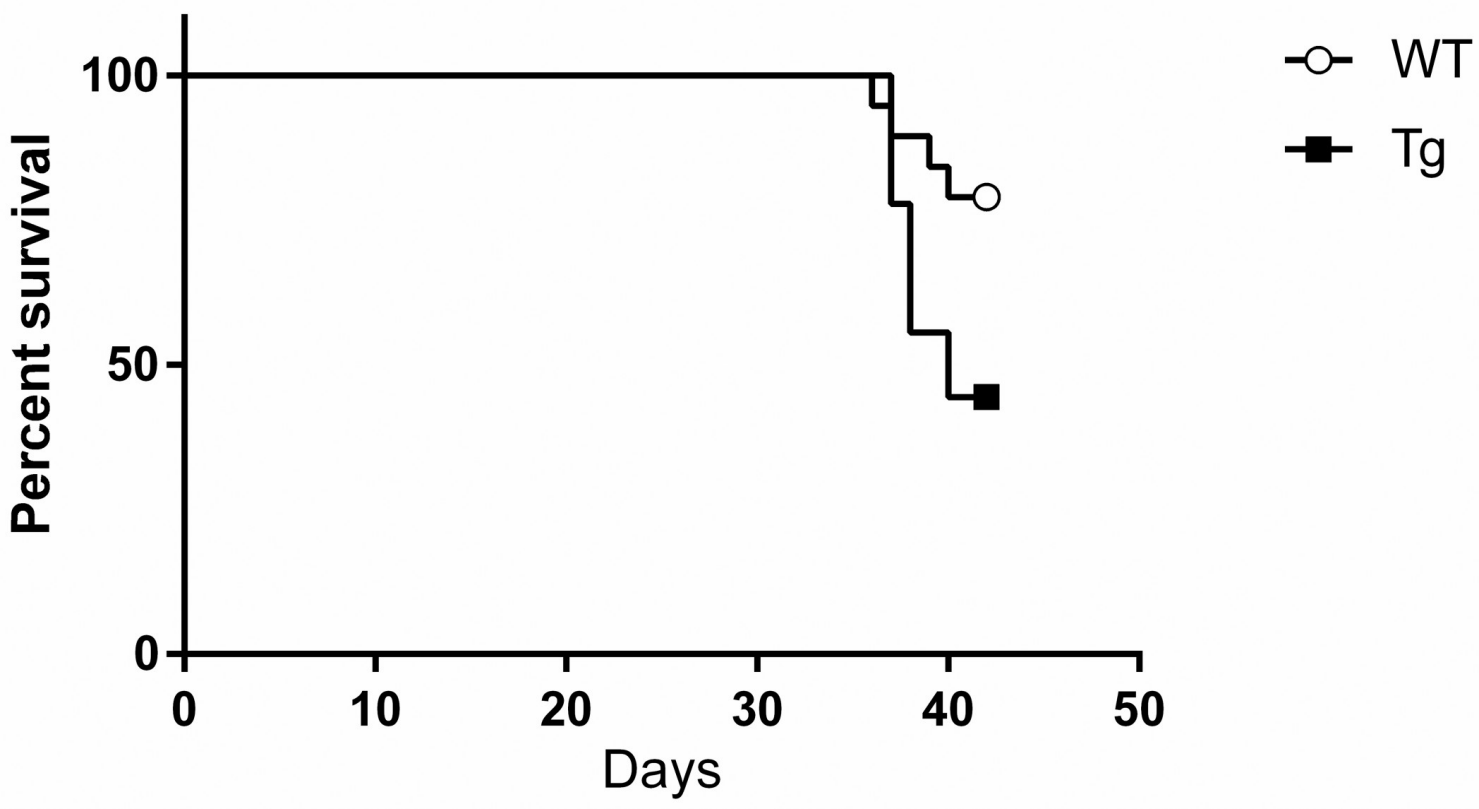
Animal survival. CTRP3 Tg mice trended towards a reduced rate of survival compared with that of WT mice (78% in WT versus 44% in CTRP3 Tg, p = 0.07). Survival data was compared by the Mantel-Cox test; n = 18 and 9, WT and Tg respectively. Abbreviations: CTRP3, C1q tumor necrosis factor-related protein-3; Tg transgenic overexpression of CTRP3; WT, Wildtype.

Animals in both groups were found dead, or culled due to moribundity (inability to stand upright), with no statistical differences between the two groups (p = 0.07, Fig 2); the data trended towards an increased mortality in the Tg mice. In total, 4 mice were culled due to moribundity (2 Tg and 2 WT) and 5 mice were found dead on daily inspection (3 Tg and 2 WT). Tissue from mice that were culled were included in additional analysis, but further analysis from mice that were found dead were not examined, as time of death could not be controlled. Overall there was a 78% and 44% survival rate in the WT and Tg groups, respectively.

### CTRP3 did not attenuate ethanol-induced hepatic lipid accumulation in female mice

**Fig 3:**
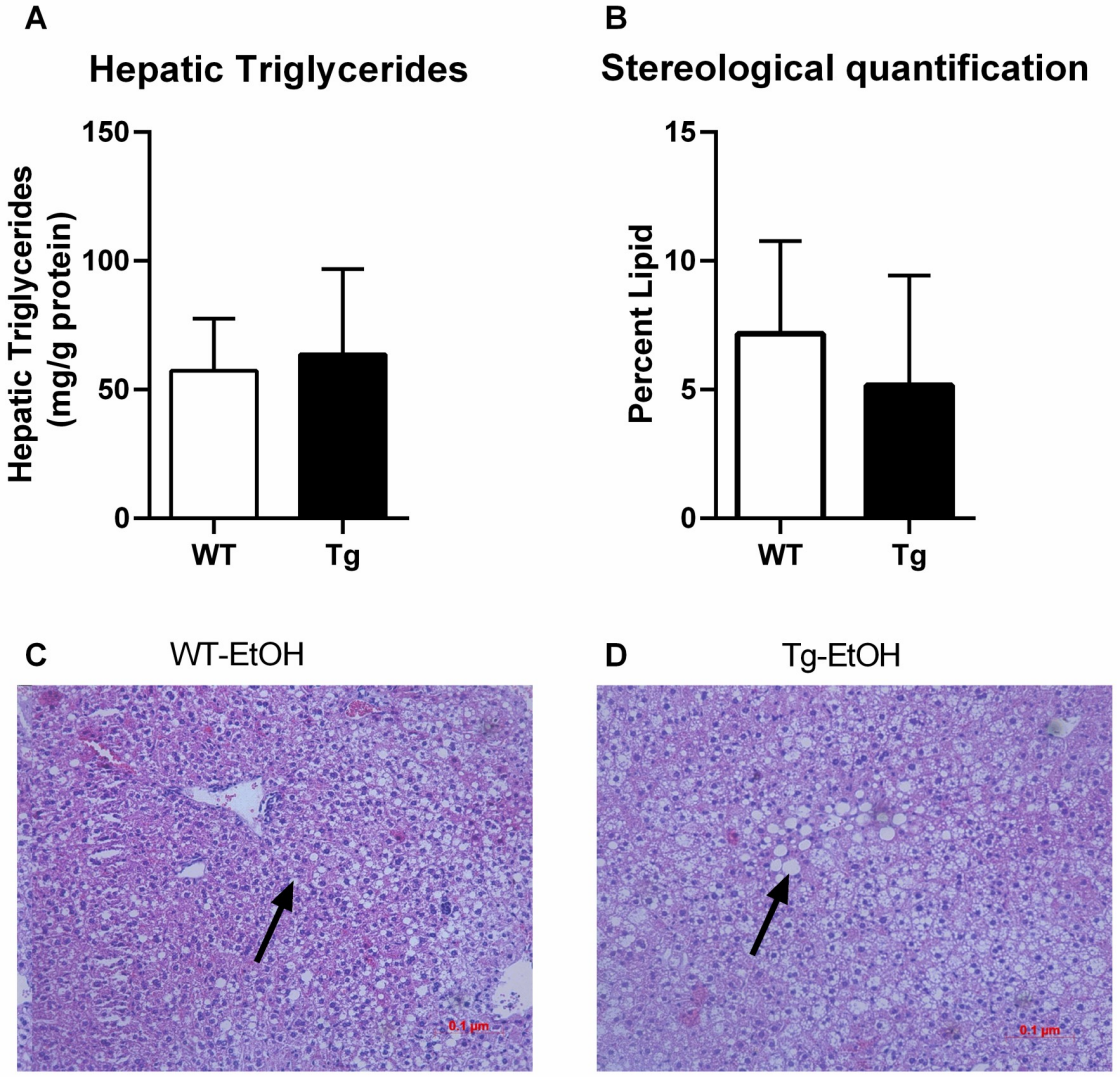
Hepatic lipid accumulation. Hepatic triglyceride levels were quantified and reported normalized to the total protein concentration (A). In addition, stereological analysis was performed on hematoxylin and eosin-stained images, 20x magnification (B). Representative hematoxylin and eosin-stained images from liver sections are also shown, the arrow indicates representative lipid droplet (*C-D*). Data are reported as means ± SD and significance was tested with an unpaired t-test; n = 10 and 5, WT and Tg respectively. Abbreviations: CTRP3, C1q tumor necrosis factor-related protein-3; Tg transgenic overexpression of CTRP3; WT, Wildtype.

Previous work demonstrated that CTRP3 prevents hepatic lipid accumulation in response to both high fat diet as well as in response to chronic alcohol feeding in male mice [19, 20]. Therefore, hepatic lipid accumulation was determined in chronically ethanol fed female mice by both quantifying hepatic triglyceride accumulation (Fig 3A) as well as via stereological analysis (Fig 3B–3D). There was no difference in lipid accumulation as determined via either method of analysis between the Tg and WT mice.

### CTRP3 did not diminish inflammatory cytokine levels in female mice

**Fig 4:**
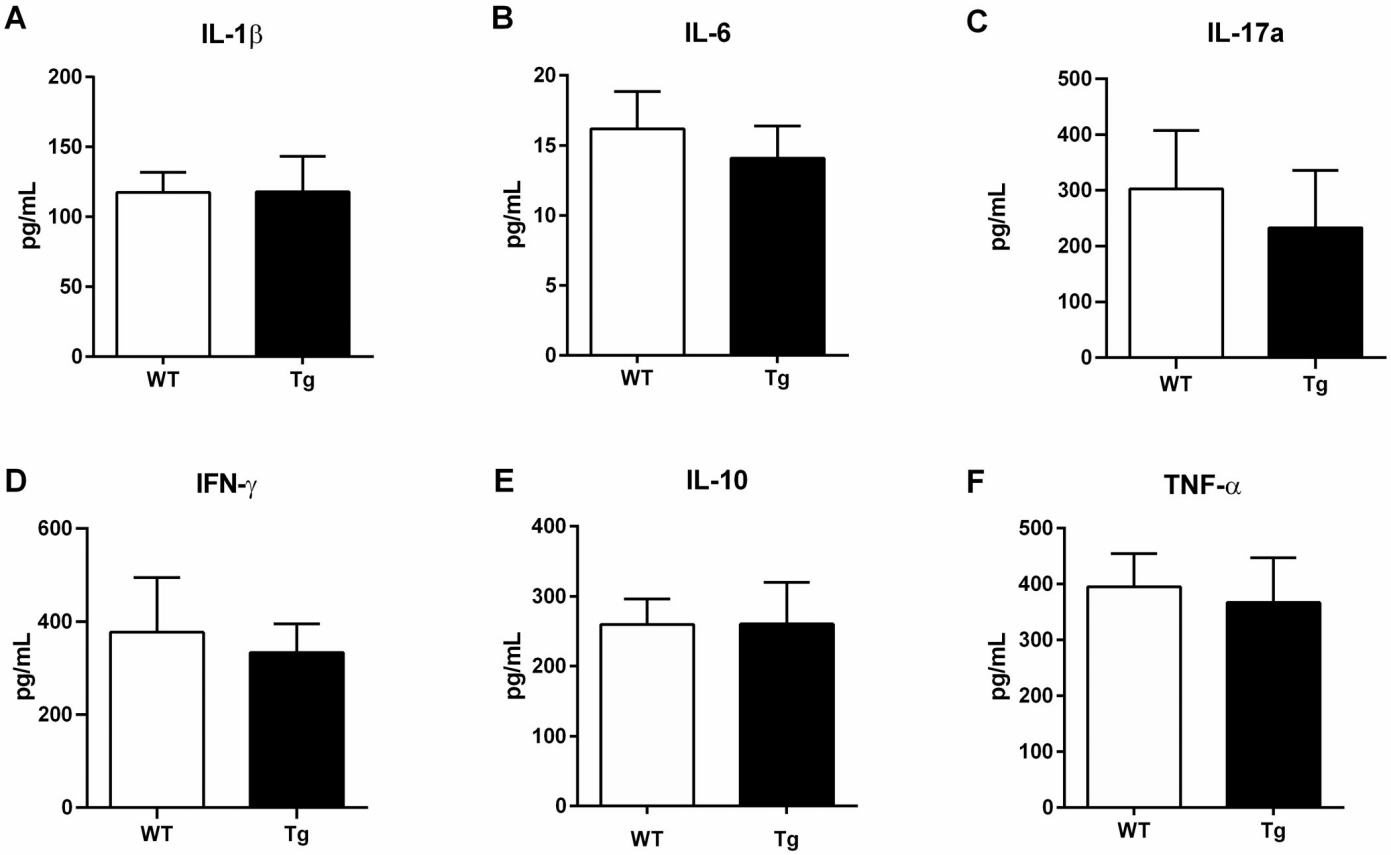
Circulating cytokines. Concentrations of circulating cytokines levels in chronic ethanol fed WT and Tg female mice were measured by Bio-Plex Multiplex assay (*A–F*). Data are reported as means + SD and significance was tested with an unpaired t-test; n = 10 and 5, WT and Tg respectively. Abbreviations: CTRP3, C1q tumor necrosis factor-related protein-3; Tg transgenic overexpression of CTRP3; WT, Wildtype; IL-1β, Interleukin 1-Beta; IL-6, Interleukin-6; IL-17α, Interleukin-17 alpha; IFN-g, Interferon-gamma; IL-10, Interleukin-10; TNF-α, Tumor necrosis factor-alpha.

To determine whether overexpression of CTRP3 would reduce overall ethanol-induced inflammation in female mice, serum cytokines were measured (Fig 4). No statistically significant differences in interleukin 1-Beta (IL-1β), Interleukin 6 (IL-6), Interleukin 17-alpha (IL-17α), Interferon gamma (IFN-g), Interleukin 10 (IL-10), or Tumor necrosis factor-alpha (TNF-α) between WT and Tg were identified (Fig 4).

### Hepatic MAPK 1/3 and PKB

**Fig 5:**
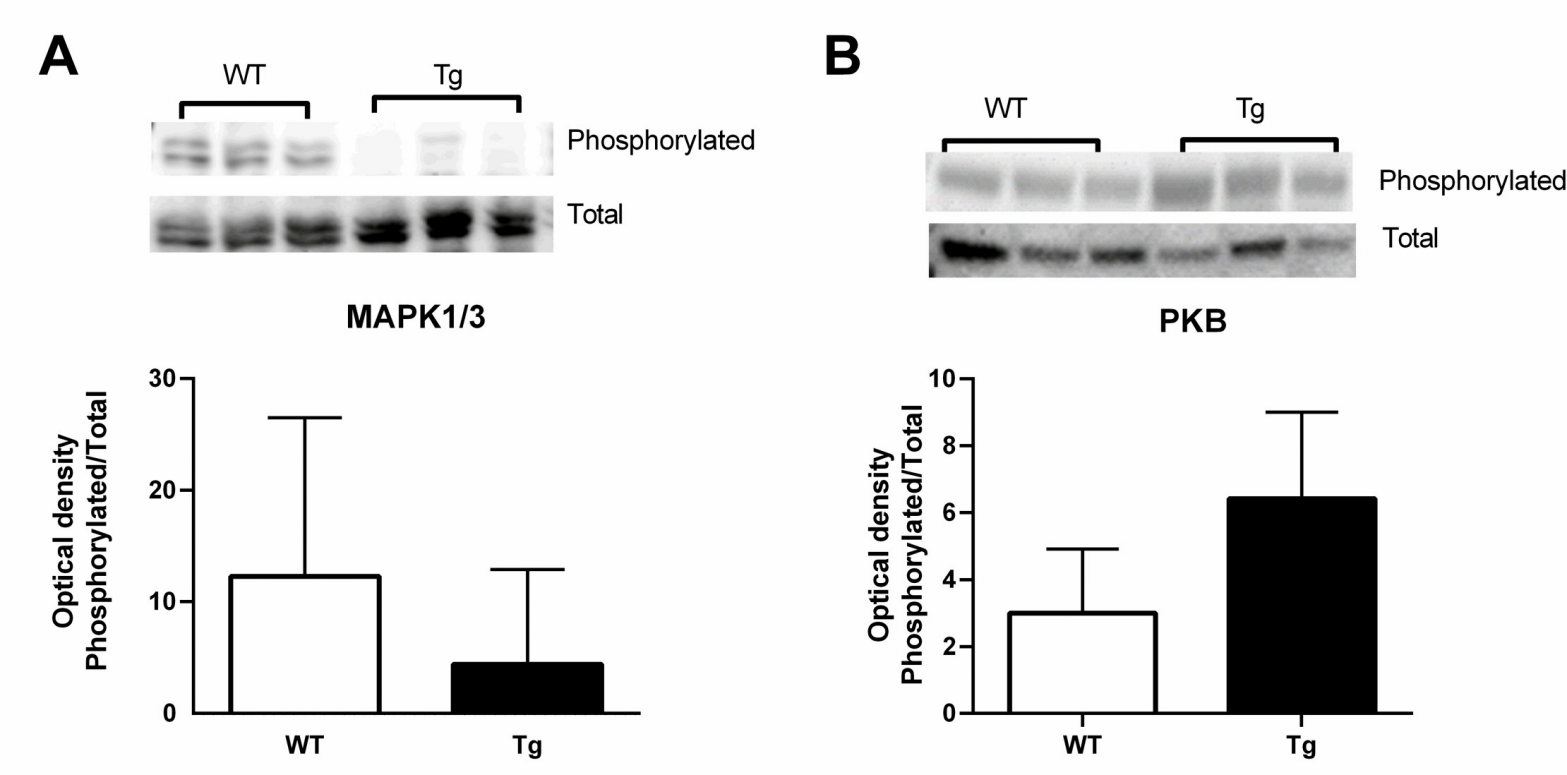
Effect of CTRP3 on protein signaling pathways. Hepatic phosphorylation status of MAPK1/3 (A) and PKB (B) were determined in chronically ethanol fed female mice with and without CTRP3 transgenic overexpression. Representative images are shown. Data are reported as means + SD and significance was tested with an unpaired t-test; n = 4–8. Abbreviations: CTRP3, C1q tumor necrosis factor-related protein-3; Tg transgenic overexpression of CTRP3; WT, Wildtype; MAPK1/3, Mitogen-activated protein kinases 1 and 3; PKB, Protein kinase B.

CTRP3 has been demonstrated to alter hepatic signaling pathways [19, 20, 31–34], specifically Mitogen-activated protein kinases 1 and 3 (MAPK1/3), also known as Extracellular signal-regulated kinase 1/2, as well as RAC-alpha serine/threonine-protein kinase, also known as Protein kinase B or Akt, hereafter referred to as PKB. Therefore, we anticipated that overexpression of CTRP3 would change the phosphorylation status of MAPK1/3 and/or PKB in the ethanol fed female mice. Although there was a trend towards reduced phosphorylation status of MAPK1/3 and an increased phosphorylation of PKB, these differences did not reach significance (Fig 5).

## Discussion

The main purpose of this study was to determine whether transgenic overexpression of CTRP3 would prevent hepatic lipid accumulation in female mice. Our lab previously demonstrated that CTRP3 overexpression significantly attenuated hepatic lipid accumulation in male mice [20]. Similar to male mice, transgenic overexpression of CTRP3 did not alter the overall food intake or body weight of the mice. However, the findings of this study are contrary to our initial hypothesis, as CTRP3 overexpression provided no benefit towards attenuating alcohol-induced hepatic lipid accumulation in female mice. We also found no changes to circulating cytokine levels or phosphorylation status of PKB or MAPK1/3, which were reported in the male mice [20]. Further, while chronic ethanol feeding results in moribundity/mortality in both male and female mice, in our previous study we found zero moribundity in CTRP3 overexpressing male mice, compared with ~30% moribundity in the WT male mice. Therefore, we also expected that CTRP3 would prevent moribundity in the female mice. However, the secondary finding of this study found no attenuation of overall moribundity with CTRP3 overexpression. Although not statistically significant, the mortality data trended towards an increased moribundity/mortality in the alcohol fed female Tg mice. Combined these data demonstrate that the protective effects of CTRP3 previously observed are sex specific and overexpression of CTRP3 is not protective for ALD or alcohol-induced moribundity in female mice.

Liver cirrhosis is the 10th leading cause of mortality in the United States; more than 40% of all cirrhosis-associated deaths are related to alcohol [8]. In 1977 Ashley et al., first documented female alcoholic patients demonstrated increased alcohol-induced morbidity (i.e. alcoholic cirrhosis) compared with male alcoholic patients [21]. These findings have been independently confirmed multiple times throughout the literature [11, 17, 22–28]. Even after drinking is ceased, females have a worse prognosis than males, indicating sexual dimorphism in the long-term effects of chronic alcohol abuse [27, 28]. Establishing the mechanism for the increased morbidity in females will uncover novel therapeutic intervention strategies to treat or manage alcohol-induce disease. Adipose tissue is the source of several secreted humoral factors (collectively called adipokines) that enter the circulation, and demonstrate a wide-range of pro- or anti-inflammatory properties. Specifically, we found that circulating CTRP3 levels were reduced with chronic alcohol exposure, but only in female mice [17]. Combined with our previous study which demonstrated that CTRP3 overexpression attenuated ALD in male mice [20], we anticipated a similar outcome with female mice. However, the data from this study demonstrates a clear sex specific difference in response to CTRP3 overexpression.

The reasons for the sexually dimorphic response to CTRP3 overexpression with chronic alcohol consumption are unknown. Our previous study, in male mice, demonstrated that CTRP3 was protective in the chronically alcohol fed model, but not in the chronic plus binge model which resulted in increased acute injury (elevated circulating cytokines and liver enzymes). It is likely that the reduction in hepatic lipid accumulation with CTRP3 overexpression is due to the observed effects of CTRP3 on increasing hepatic lipid oxidation [19], and does little to protect the hepatocytes directly from the alcohol-induced injury. Although the amount of alcohol consumption is generally related to the severity of the disease, females are documented to have a higher propensity for alcoholic morbidity compared with males with a lower total and relative amount of alcohol consumption [11, 21, 22, 24]. Alcohol consumption increases intestinal gut permeability thus causing elevation in endotoxins delivered to the liver and disrupts gut-derived hormones which interferes with hepatic signaling and metabolic control [35]. Female sex hormones have been linked to increased alcohol-induced portal endotoxin levels [36, 37]. Further, female sex hormones indirectly increase Kupffer cell (liver resident macrophages) sensitivity to endotoxins [25, 38–40]. Lastly, females also have reduced alcohol dehydrogenase (ADH) activity, which in turn increases the proportion of ingested alcohol that enters the hepatic portal system [29]. As shown in our previous study, the 6-week alcohol-feeding protocol results in cytokines levels that are approximately 3 times higher in female mice compared with alcohol fed male mice [17]. Combined these data suggest that the increased inflammation caused by the alcohol exposure protocol in female mice could prevent any potential beneficial effects of CTRP3.

### Future directions

It should be established whether female sex hormones interfere with the actions of CTRP3 directly or whether the ineffectiveness of CTRP3 in preventing ALD in females is due to the suspected increased effect of alcohol in females [11, 21, 22, 24]. Further, High fat diet-induced non-alcoholic fatty liver disease (NAFLD), results in hepatic steatosis without injury and CTRP3 overexpression has been identified to prevent NAFLD in male mice [19]. Therefore, determining whether CTRP3 overexpression prevents NAFLD in female mice should be explored. Additionally, repeating the alcohol feeding exposures experiments plus/minus CTRP3 overexpression in male mice supplemented with estrogen and/or ovariectomized female mice would help to establish whether estrogen directly interferes with CTRP3’s hepatic functions.

### Study limitations

Based on previous work we anticipated a high mortality of the WT mice with little to no mortality in the Tg mice [31]. To reduce overall mouse moribundity/mortality modifications to the protocol were implemented as suggested by Gao et al. [1]. Specifically, a heat pad was placed on the side of all cages to increase the ambient temperature while still allowing the mice to self-regulate (i.e., move to the unheated or heated side as desired) and a secondary alternative ethanol-free water source was provided. These modifications significantly increased the survival of the WT mice compared with our previous study [31]. However, as we anticipated lower mortality in the CTRP3 Tg mice we started with a n = 9, and even though more than 50% of the Tg group did not survive the difference was not statistically significant (p = 0.07). Although increasing the group size may have demonstrated significance, our initial hypothesis was that CTRP3 overexpression would be protective and the results of this study clearly indicated that our hypothesis was not supported.

## Supporting information

S1 Fig Supporting images for Fig 5. Full ponceau red staining and immunoblots used to generate the data for Fig 5. Abbreviations: CTRP3, C1q tumor necrosis factor-related protein-3; Tg, transgenic overexpression of CTRP3; WT, Wildtype; MAPK1/3, Mitogen-activated protein kinases 1 and 3; PKB, Protein kinase B.
(TIF)

S2 Fig Circulating CTRP3 levels. Circulating CTRP3 levels in wildtype compared with transgenic male and female mice. A) Ponceau red staining and B) immunoblot using an anti-mouse CTRP3 on the left (R&D systems Cat # AF2436). Abbreviations: Tg, transgenic overexpression of CTRP3; WT, Wildtype.
(TIF)
